# Predicting Group‐Based Trajectories of Oral Health‐Related Quality of Life From Late Adolescence to Early Adulthood Using K‐Means Clustering Algorithm

**DOI:** 10.1111/jphd.70007

**Published:** 2025-09-16

**Authors:** Chukwuebuka Ogwo, Grant Brown, John Warren, Erliang Zeng, Murray Thomson, Steven Levy

**Affiliations:** ^1^ Department of Oral Health Policy and Epidemiology Harvard University School of Dental Medicine Boston Massachusetts USA; ^2^ Department of Biostatistics, College of Public Health The University of Iowa Iowa City Iowa USA; ^3^ Department of Preventive and Community Dentistry The University of Iowa College of Dentistry Iowa City Iowa USA; ^4^ Division of Biostatistics and Computational Biology The University of Iowa College of Dentistry Iowa City Iowa USA; ^5^ Department of Oral Sciences, Sir John Walsh Research Institute, Faculty of Dentistry University of Otago Dunedin New Zealand; ^6^ Department of Epidemiology, College of Public Health The University of Iowa Iowa City Iowa USA

**Keywords:** K‐means, longitudinal data, machine learning, oral health‐related quality of life, prediction, trajectories

## Abstract

**Objectives:**

To identify and analyze the patterns of change in Oral Health‐Related Quality of Life (OHRQoL) from late adolescence to early adulthood (ages 17 to 23) using a machine learning algorithm.

**Methods:**

This longitudinal trajectory study used data from the Iowa Fluoride Study (IFS). Participants were recruited at birth from eight Iowa hospitals between 1992 and 1995. OHRQoL was assessed at ages 17, 19, and 23 using three validated questionnaires: the Child Perceptions Questionnaire (CPQ_11‐14_), Global Oral Health Rating (GOHR), and Visual Analog Scale of Quality of Life (VisQoL). Of the 437 individuals assessed at age 17, 402 were re‐assessed at age 19 and 367 at age 23 (91% retention rate). The K‐Means for Longitudinal data (KmL) algorithm was applied to identify distinct trajectory groups for each measure. Associations between trajectory group membership and sociodemographic variables were examined using logistic regression. All analyses were performed in R (version 4.1.3).

**Results:**

Two distinct trajectory groups were identified for each OHRQoL measure, representing consistently better versus persistently worsening oral health experiences. For the CPQ_11‐14_, 84.8% of participants were in the “Favorable” group and 15.2% in the “Unfavorable” group. GOHR classified 57.2% as “Favorable” and 42.8% as “Unfavorable,” while VisQoL showed 67.5% and 32.5%, respectively. Despite differing proportions, all instruments reflected similar directional trends. Higher socioeconomic status was associated with favorable trajectory group membership (*p* < 0.05).

**Conclusion:**

Most participants followed a favorable OHRQoL trajectory and were from higher socioeconomic backgrounds. These findings highlight the value of longitudinal, multi‐measure approaches in identifying at‐risk subgroups.

## 1 Introduction

Oral Health‐Related Quality of Life (OHRQoL) is a central construct in patient‐centered care in dentistry. According to the Surgeon General's report Oral Health in America, OHRQoL “reflects people's comfort when eating, sleeping, and engaging in social interaction; their self‐esteem; and their satisfaction concerning their oral health” [1]. Oral health conditions affect both physical and psychological well‐being, influencing how individuals grow, look, speak, chew, taste food, and socialize [2]. In clinical settings, consideration of OHRQoL can facilitate decision‐making regarding treatment risks, benefits, and costs. For instance, children aged 5–16 years with very high caries rates who were treated under general anesthesia had significantly greater improvements in OHRQoL compared to those treated over multiple clinical visits [3]. Understanding patients' perceptions of their oral health needs enables dentists to tailor preventive and treatment strategies that improve quality of life and optimize resource use [2, 4].

At the population level, oral health conditions are associated with measurable losses in quality‐adjusted life expectancy (QALE). In a national study of US adults, dental caries alone were linked to a mean loss of 0.43 years in QALE, increasing to 8.15 years when periodontitis and malocclusion were also considered [5]. This study also documented disparities in QALE loss across the life course, with individuals having a high school education or less experiencing greater loss compared to college graduates. Such findings highlight that improvements in oral health can have large impacts on overall well‐being.

Despite the clear importance of OHRQoL, little is known about its longitudinal changes during the transition from adolescence to early adulthood, a developmental stage when oral health behaviors, social roles, and self‐image undergo significant changes. While a small number of longitudinal studies have been conducted [6, 7, 8, 9, 10], none have specifically examined OHRQoL trajectories over this period. Most existing research focuses on younger adolescents, with three studies involving participants aged 12 [7, 8, 9], one including children aged 6 to 16 [6], and another involving participants aged 11 to 14 [10]. Collectively, these studies found that factors such as greater emotional well‐being [6], lower dental caries experience [7, 8, 9], and higher parental education [8] were associated with improved OHRQoL. However, their scope was limited in both age range and analytical approach, and outcomes such as orthodontic or esthetic treatment compliance, while relevant, do not fully capture the diversity of OHRQoL patterns over time.

Trajectory analysis offers a way to characterize the course of measured variables and identify subgroups of individuals with similar patterns. To address the gaps in both longitudinal and trajectory‐based OHRQoL research, we applied K‐means for Longitudinal Data, a machine learning method that accommodates high‐dimensional and noisy longitudinal data [11, 12]. This approach allows for more precise identification of distinct OHRQoL patterns than traditional statistical techniques. Therefore, the objective of this study was to identify group‐based trajectories of OHRQoL from late adolescence (age 17) to early adulthood (age 23) in the Iowa Fluoride Study cohort. We hypothesized that multiple distinct trajectory groups would emerge, each reflecting different patterns of change in OHRQoL over time.

## 2 Methods

This study assessed trajectories of OHRQoL from late adolescence to early adulthood using Iowa Fluoride Study (IFS) data, a prospective cohort study of dental caries, dental fluorosis, fluoride exposure, and other factors that also assessed OHRQoL beginning in the 1990s. Sampling was convenience‐based; participants were recruited from eight Iowa hospitals selected to represent the state's geographic and socioeconomic diversity, including urban, suburban, and rural locations. Hospitals were chosen based on delivery volume (> 500 births/year) and payer mix data from public health records to ensure broad coverage. The cohort captured approximately 65% of Iowa's annual births during the 1992 to 1995 recruitment period, with follow‐up for 23 years via oral health questionnaires and examinations [13].

The University of Iowa Institutional Review Board approved all study components and procedures. Informed consent was obtained from parents before the age 17 assessment (with adolescent assent), and directly from participants at ages 19 and 23. At enrollment, researchers collected extensive baseline contact information, including alternate contacts, and implemented structured follow‐up through clinic visits and mailed questionnaires. Retention strategies included phone calls and collaboration with community networks to locate participants. Follow‐up assessments were conducted either in person during study visits or through self‐administered mailed surveys; telephone interviews were used for participants unable to attend in person [13].

Three validated OHRQoL instruments were administered at ages 17, 19, and 23: the Child Perceptions Questionnaire (CPQ_11‐14_), the Global Oral Health Rating (GOHR), and the Visual Analog Rating of the Quality of Life (VisQoL) [14, 15, 16, 17]. The use of multiple instruments was intentional to capture complementary aspects of OHRQoL: CPQ_11‐14_ provides domain‐specific detail on oral symptoms, functional limitations, emotional well‐being, and social well‐being; GOHR offers a concise global rating sensitive to perceived treatment outcomes; and VisQoL provides a continuous analog scale for small but meaningful perceived changes. Using these tools in combination allowed for triangulation, enhanced measurement reliability, and comparability with adolescent and young adult studies.

While the Oral Health Impact Profile (OHIP) is the most widely used OHRQoL measure for adult populations, it was not adopted here because it is less sensitive to developmental and psychosocial changes relevant during the transition from adolescence to adulthood. Introducing OHIP only at later waves would have reduced longitudinal comparability with earlier assessments. Although CPQ_11‐14_ was originally developed for children and younger adolescents, prior research supports its validity and reliability in young adult populations [18], making it suitable for ages 19 to 23 when continuity of measurement is a priority. Maintaining the same instrument across waves ensured observed changes reflected real developmental and experiential differences rather than measurement artifacts.

The CPQ_11‐14_ contains 37 items in four domains: oral symptoms [5], functional limitations [9], emotional well‐being [10], and social well‐being [19]. Each item is scored on a 5‐point scale from “Never” = 0 to “Very often” = 4; lower total scores indicate better OHRQoL [15]. The GOHR consists of two questions scored 1–5, summed for a total score of 2–10, with lower scores indicating better OHRQoL [16]. The VisQoL is a single‐item visual analog scale from 0 (“worst oral health imaginable”) to 100 (“perfect” oral health). Inclusion for trajectory analysis required completion of OHRQoL questionnaires at least twice across the three time points.

## 3 Statistical Analysis

The dataset, including psychosocial (OHRQoL) and demographic variables, was extracted from the IFS database; analyses were performed in R (Version 4.1.3). Descriptive statistics (frequency, mean, median, maximum, minimum, quartiles, interquartile range) were calculated for individual and group‐level OHRQoL scores.

Trajectory analysis used the unsupervised K‐Means for Longitudinal data (KmL) algorithm [20]. Participants with data for at least two waves were included (*n* = 374 for CPQ_11‐14_/GOHR; *n* = 369 for VisQoL). Missing data for a single timepoint were imputed using linear interpolation, preserving trajectory shapes via Euclidean distance with Gower's adjustment [21]. This approach retains global maximum identification advantages over deterministic clustering. After clustering, trajectories were visualized using KmL's graphical interface [11].

The Calinski and Harabasz criterion [22] was used to assess model performance; higher values indicate better cluster separation. Associations between trajectory group membership and demographic variables were tested using Pearson's chi‐square for categorical predictors (sex, socioeconomic status) and independent samples *t* tests for continuous variables.

Socioeconomic status (SES) was categorized as low, middle, or high based on parental education and household income: low (< 130% federal poverty level [FPL] or ≤ high school education), middle (130%–300% FPL or some college), and high (> 300% FPL or ≥ bachelor's degree), following US public health standards [23]. Logistic regression assessed multivariable associations, with α = 0.05. Although a formal a priori power calculation was not feasible due to the longitudinal design, the achieved sample sizes exceed those recommended for detecting medium effect sizes in trajectory‐based analyses, supporting adequacy for the study objectives.

## 4 Results

The number of IFS participants who completed the oral health‐related quality of life (OHRQoL) questionnaires at ages 17, 19, and 23 is summarized as follows. For the CPQ_11‐14_ questionnaire, the numbers were 412 at age 17, 331 at age 19, and 345 at age 23. For the GOHR questionnaire, 341, 311, and 346 participants completed the questionnaire at the same respective ages. For the VisQoL questionnaire, the numbers were 403, 331, and 346, respectively. After applying the inclusion criteria for trajectory analysis, the final analytic samples included 374 participants for CPQ_11‐14_, 374 participants for GOHR, and 369 participants for VisQoL. The percentage of missing data was 9.2% for CPQ11‐14, 8.8% for GOHR, and 8.4% for VisQoL.

Overall, there were 44% males and 56% females in the study cohort. With respect to socioeconomic status (SES), 13% of participants came from low SES families, 33% from middle SES families, and 53% from high SES families. In terms of racial and ethnic background, more than 95% of the participants identified as non‐Hispanic whites.

### 4.1 Descriptive OHRQoL Scores

As presented in Table **1**, the mean CPQ11‐14_11–14_ scores across the three assessment points were 9.02 at age 17, 8.33 at age 19, and 9.72 at age 23. These values indicate relatively low levels of oral health‐related impact overall, although there was a slight increase from age 19 to age 23. Among the subdomains of the CPQ_11‐14_, oral symptoms had the highest mean scores at all three time points: 4.17 at age 17, 4.13 at age 19, and 4.64 at age 23. This pattern suggests that oral symptoms contributed the most to the total CPQ_11‐14_ scores compared to other subscales such as functional limitations, emotional well‐being, or social well‐being.

**TABLE 1:** Summary statistics for the three OHRQoL variables.

	Age	*N*	Mean	SD	Median	Min	Max
Child Perception Questionnaire (CPQ)	17	412	9.02	9.34	6	0	68
	19	331	8.33	8.24	6	0	47
	23	345	9.05	9.72	6	0	85
CPQ—Oral Symptoms	17	413	4.17	2.64	4	0	14
	19	331	4.13	2.60	4	0	14
	23	346	4.64	3.03	4	0	18
CPQ—Functional Limitations	17	413	1.55	2.67	0	0	21
	19	331	1.24	2.39	0	0	15
	23	345	1.37	2.61	0	0	19
CPQ—Emotional WellBeing	17	412	2.22	4.31	0	0	28
	19	331	2.17	4.17	0	0	25
	23	346	2.22	4.11	0	0	31
CPQ—Social WellBeing	17	412	1.08	2.67	0	0	24
	19	331	0.79	2.14	0	0	17
	23	346	0.81	2.43	0	0	23
Global Oral Health Rating (GOHR)	17	341	3.92	1.45	4	2	10
	19	311	3.93	1.42	4	2	8
	23	313	4.22	1.45	4	2	9
Visual Scoring of Quality of Life (VisQoL)	17	403	85.99	12.35	90	20	100
	19	331	84.24	13.34	87	25	100
	23	346	83.69	13.73	85	25	100

*Note:* The higher the score, the worse the CPQ and GOHR; the lower the score, the worse the VisQol. CPQ possible range = 0 to 148; GOHR possible range = 2 to 10; VisQoL possible range = 0 to 100.

The mean scores for the GOHR measure were relatively stable over time, with values of 3.92 at age 17, 3.93 at age 19, and 4.22 at age 23. This suggests a relatively consistent perception of general oral health‐related quality of life during this developmental period, with only a slight increase in perceived impact over time. VisQoL scores, which reflect visual oral health‐related quality of life with higher scores indicating better quality of life, showed a modest downward trend over time. The average VisQoL scores were 85.99 at age 17, 84.24 at age 19, and 83.69 at age 23, indicating a gradual decrease in perceived visual oral health‐related quality of life across late adolescence and early adulthood.

### 4.2 Trajectory Analysis of OHRQoL

**FIGURE 1:**
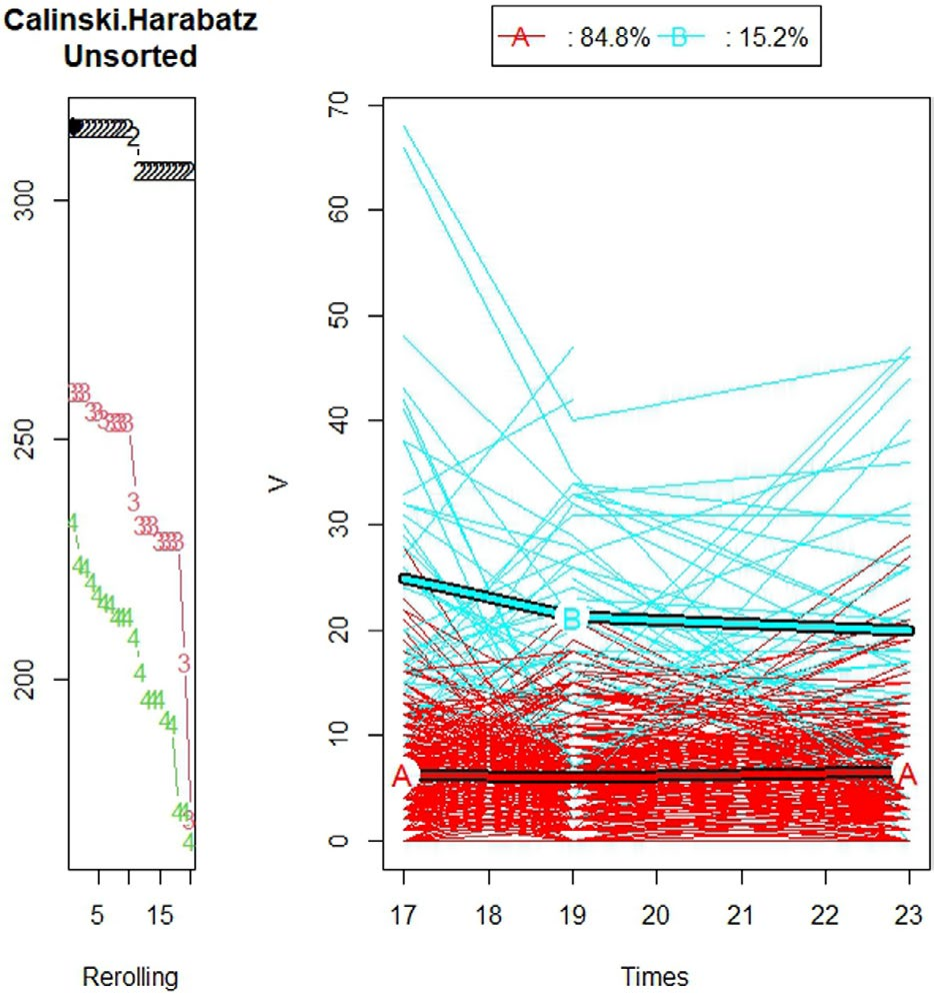
Plot showing the Calinski–Harabasz scores for the different numbers of trajectory groups and individuals' CPQ trajectories and the trajectory of their centroids (for individuals with a maximum of 1 missing time point) (*N* = 374). [Color figure can be viewed at wileyonlinelibrary.com]

**FIGURE 2:**
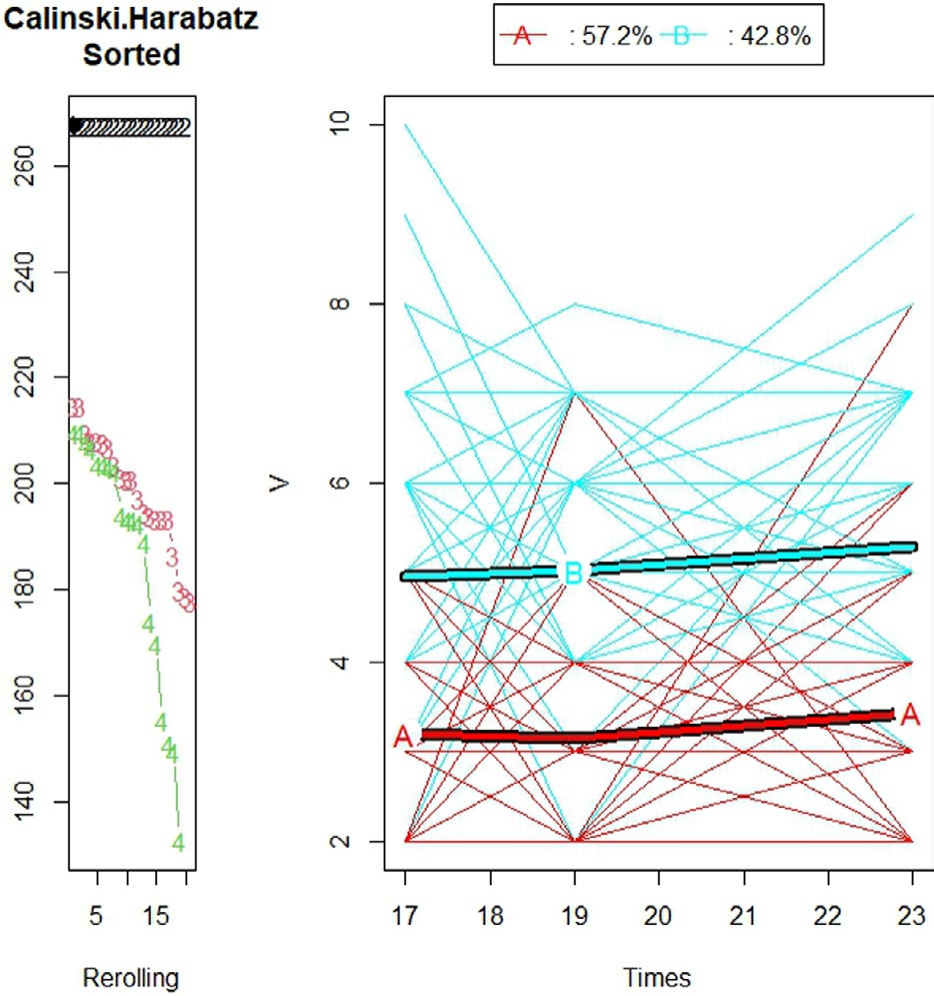
Plot showing the Calinski–Harabatz scores for the different numbers of trajectory groups and individuals' GOHR trajectories and the trajectory of their centroids (for individuals with a maximum of 1 missing time point) (*N* = 374). [Color figure can be viewed at wileyonlinelibrary.com]

**FIGURE 3:**
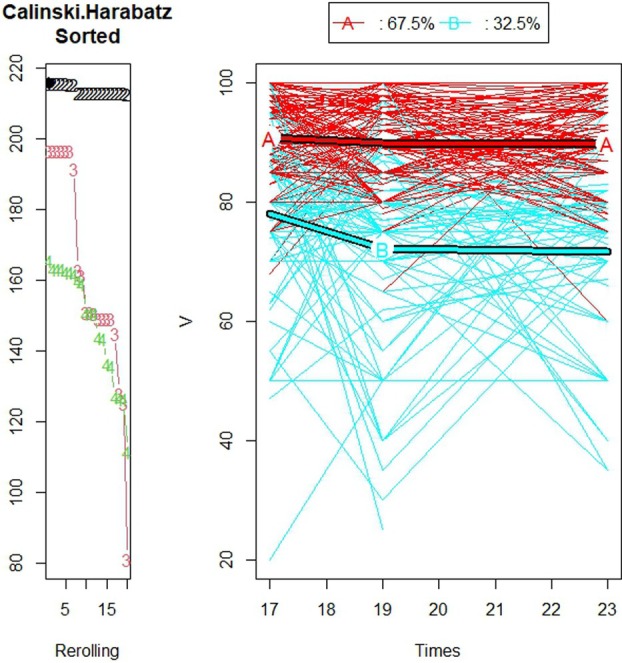
Plot showing the Calinski–Harabatz scores for the different numbers of trajectory groups and individuals' VisQoL trajectories with the trajectory of their centroids (for individuals with a maximum of 1 missing time point) (*N* = 369). [Color figure can be viewed at wileyonlinelibrary.com]

The K‐means for longitudinal data (KmL) algorithm found two trajectory groups to be the optimal number of partitions for each of the three OHRQoL measures: CPQ_11‐14_, GOHR, and VisQoL (Caliński‐Harabasz values = 315, 267, 215, respectively) (Figure 1). Figure 1 shows a graphical representation of the two trajectory groups for CPQ_11‐14_ and the percentage of individual trajectories in each group. Favorable trajectory group are individuals with consistently better OHRQoL scores over time. Unfavorable trajectory group is individuals with poorer OHRQoL scores over time. Three hundred and seventeen (84.8%) individual trajectories comprised trajectory group A (Favorable OHRQoL trajectory group) and 57 (15.2%) were in trajectory group B (Unfavorable OHRQoL trajectory group). Figure 2 shows a graphical representation of the two trajectory groups for GOHR and the percentages of individual trajectories in each group. Two hundred and fourteen (57.2%) of the individual trajectories were in the favorable OHRQoL trajectory group, and 160 (42.8%) were in the unfavorable OHRQoL trajectory group. Figure 3 shows a graphical representation of the two trajectory groups for VisQoL and the percentages of individual trajectories in each group. Two hundred and forty‐nine (67.5%) of the individual trajectories were in the favorable trajectory group, and 120 (32.5%) were in the unfavorable trajectory group. The trajectory analysis for 3 groups is also reported for all the OHRQoL measures in Appendix 1: Supporting Information, including the medium OHRQoL trajectory group.

Table 2 summarizes the mean OHRQoL at ages 17, 19, and 23 for each of the trajectory groups for all three OHRQoL measures. The mean CPQ_11‐14_ scores at ages 17, 19, and 23 for the favorable trajectory group were 6.3, 5.9, and 6.5, respectively, while the mean CPQ_11‐14_ scores for the unfavorable trajectory group were 24.88, 21.29, and 20.00, respectively. The mean GOHR scores at ages 17, 19, and 23 for the favorable trajectory group were 3.20, 3.14, and 3.43, respectively, while the mean GOHR scores for the unfavorable trajectory group were 4.94, 5.02, and 5.29, respectively. The mean VisQoL scores at ages 17, 19, and 23 for the favorable trajectory group were 90.74, 90.00, and 89.81, respectively, while the mean VisQoL scores for the unfavorable trajectory group were 78.04, 72.13, and 71.66, respectively. There were statistically significant differences between trajectory groups for all three OHRQoL mean scores (*p* = 0.008, *p* < 0.001, and *p* = 0.01, respectively).

**TABLE 2:** Mean OHRQoL scores at ages 17, 19, and 23 and the percentage distributions for the trajectory groups.

	Mean CPQ score (*N* = 374)			Percentage distribution
	Age 17	Age 19	Age 23	
Trajectory group A	6.33	5.94	6.52	84.8%
Trajectory group B	24.88	21.29	20.00	15.2%

	Mean GOHR score (*N* = 374)			Percentage distribution
	Age 17	Age 19	Age 23	
Trajectory group A	3.20	3.14	3.43	57.2%
Trajectory group B	4.94	5.02	5.29	42.8%

	Mean VisQoL score (*N* = 369)			Percentage distribution
	Age 17	Age 19	Age 23	
Trajectory group A	90.74	90.00	89.81	67.5%
Trajectory group B	78.04	72.13	71.66	32.5%

Abbreviations: CPQ = Child Perception Questionnaire; GOHR = Global Oral Health Rating; VisQoL = Visual Scoring of Quality of Life.

As shown in Table 3, socioeconomic status was significantly associated with OHRQoL trajectory group membership for both the CPQ_11‐14_ and VisQoL measures. There were significant differences in the OHRQoL trajectory group membership across the different categories of SES based on CPQ_11‐14_ and VisQoL (both *p* = 0.003), but not for GOHR (*p* = 0.06). There were no significant differences in trajectory group membership by sex for CPQ_11‐14_ (*p* = 1.00), GOHR (*p* = 0.23), and VisQoL (*p* = 0.93).

**TABLE 3:** Bivariate analyses showing the associations between sex and socioeconomic status and the three OHRQoL trajectory group memberships.

	CPQ trajectory group by sex		*p* a
	Male	Female	
Trajectory group A	43.8%	56.2%	1.00
Trajectory group B	43.4%	56.6%	

	GOHR trajectory group by sex		
	Male	Female	
Trajectory group A	40.7%	59.3%	0.23
Trajectory group B	47.9%	52.1%	

	VisQoL trajectory group by sex		
	Male	Female	
Trajectory group A	44.2%	55.8%	0.93
Trajectory group B	43%	57%	

	CPQ trajectory group by SES			
	Low	Middle	High	
Trajectory group A	11.9%	30.6%	57.5%	0.003
Trajectory group B	20.8%	47.9%	31.2%	

	GOHR trajectory group by SES			
	Low	Middle	High	
Trajectory group A	10.3%	31.4%	58.4%	0.06
Trajectory group B	17.6%	35.9%	46.6%	

	VisQoL trajectory group by SES			
	Low	Middle	High	
Trajectory group A	10%	32.1%	57.9%	0.03
Trajectory group B	19.4%	35.9%	44.7%	

Abbreviations: CPQ = Child Perception Questionnaire; GOHR = Global Oral Health Rating; SES = Socioeconomic status; VisQoL = Visual Scoring of Quality of Life.

^a^ Associations between sex/socioeconomic status and OHRQoL trajectory groups were tested using Pearson's chi‐square tests.

The logistic regression in Table 4 showed that keeping other variables constant, participants from high SES backgrounds had 69% lower odds of being classified in the unfavorable CPQ_11‐14_ trajectory group compared to those from low SES backgrounds. Similarly, high SES individuals had 53.3% lower odds of being in the unfavorable GOHR trajectory group and 60.1% lower odds of being in the unfavorable VisQoL trajectory group. These findings highlight the strong positive influence of higher socioeconomic status on long‐term oral health‐related quality of life during adolescence and young adulthood.

**TABLE 4:** Multivariable logistic regression showing the associations between race, sex and socioeconomic status and the three OHRQoL trajectory group memberships.

CPQ		Model 1			Model 2 (final)		
Variable		Odds ratio	Lower limit	Upper limit	Odds ratio	Lower limit	Upper limit
Sex	Female	1.03	−0.60	0.68	—	—	—
	Male (Ref)				—	—	—
Socio‐economic Status	High	0.31	−2.04	−0.25	**0.31**	−**2.05**	−**0.26**
	Middle	0.90	−0.94	0.78	0.89	−0.94	0.77
	Low (Ref)						
Akaike Information Criteria (AIC)		265.78			263.79		

GOHR		Model 1			Model 2 (final)		
Variable		Odds ratio	Lower limit	Upper limit	Odds ratio	Lower limit	Upper limit
Sex	Female	0.71	−0.80	0.12	—	—	—
	Male (Ref)				—	—	—
Socio‐economic Status	High	0.47	−1.49	−0.11	**0.47**	−**1.46**	**‐ 0.08**
	Middle	0.63	−1.20	0.26	0.59	−1.13	0.32
	Low (Ref)						
Akaike Information Criteria (AIC)		429.14			429.29		

VISQoL		Model 1			Model 2 (final)		
Variable		Odds ratio	Lower limit	Upper limit	Odds ratio	Lower limit	Upper limit
Sex	Female	1.09	−0.39	0.58	—	—	
	Male (Ref)				—	—	
Socio‐economic Status	High	0.40	−1.62	−0.21	**0.40**	−**1.62**	**‐ 0.24**
	Middle	0.59	−1.27	0.21	0.58	−1.28	0.19
	Low (Ref)						
Akaike Information Criteria (AIC)		396.68			394.80		

*Note:* Model 1 included both sex and socioeconomic status (SES) as predictors of OHRQoL trajectory groups. Model 2 (the final model) excluded **sex** due to its non‐significant association (odds ratios close to 1.0 with confidence intervals spanning null values), while retaining SES, which demonstrated statistically meaningful effects. The decision to simplify the model was supported by improved parsimony, as evidenced by lower Akaike Information Criterion (AIC) values in Model 2.

## 5 Discussion

This study examined trajectories of Oral Health‐Related Quality of Life (OHRQoL) from late adolescence to early adulthood using three distinct measures: the Child Perceptions Questionnaire (CPQ11‐14), Global Oral Health Rating (GOHR), and Visual Analog Rating of Quality of Life (VisQoL). Assessing OHRQoL trajectories in adolescence and early adulthood is important because it shows how perceived oral health changes during a formative life stage. Trajectory patterns can reveal persistent disparities that single time‐point measures might miss. This approach can help identify individuals at risk of poor long‐term outcomes and guide early, targeted interventions aimed at improving oral health and quality of life. By applying KmL, we identified two distinct trajectory groups for each measure, revealing a general decline in OHRQoL between ages 17 and 23. Despite this overall decline, participants in our cohort consistently reported more favorable OHRQoL scores than those observed in previous longitudinal studies [6, 7, 8, 9, 10].

Domain‐specific analysis of CPQ_11‐14_ scores indicated that oral symptoms were the most persistent concern, while functional impacts remained minimal. Emotional impacts demonstrated greater variability than social impacts, suggesting that oral health during this transitional period affects psychological well‐being more than social functioning. These patterns underscore the importance of symptom management and emotional support in interventions targeted toward young adults.

Our findings align with prior literature. For instance, Jaeken et al. reported similar trends in adolescent OHRQoL trajectories, though their participants had overall worse scores than ours [6]. These differences likely reflect variations in socioeconomic status (SES), as our predominantly higher‐SES cohort reported more favorable outcomes. This is consistent with Brondani et al., who found that household income trajectories significantly influenced adolescent OHRQoL [24].

However, direct comparisons with Jaeken et al. must be made cautiously. Their study focused on a younger cohort (ages 11–16) undergoing orthodontic treatment, while ours evaluated a general population cohort aged 17 to 23—a developmental stage associated with distinct psychosocial and health behaviors. The clinical focus and specific dental challenges in the orthodontic population likely introduced unique OHRQoL concerns that are not directly comparable to our more general sample.

Importantly, while most prior studies have examined OHRQoL during childhood or adolescence, few have extended into early adulthood. Our study offers novel insights into this underexplored transitional phase, which is critical for the development of long‐term oral health behaviors and overall well‐being. Moreover, by using longitudinal rather than cross‐sectional data, we captured dynamic OHRQoL changes over time, enabling identification of distinct trajectory groups that cross‐sectional designs would likely obscure. These methodological advantages further emphasize the need for caution when comparing our findings with those of previous research.

A key strength of our study is the application of an unsupervised machine learning algorithm (KmL), which is particularly suited to handling the complexities of longitudinal data and associated missingness. Previous research has demonstrated that KmL, particularly when augmented with distance metrics such as Gower's or Fréchet distance, outperforms traditional models like SAS's Proc Traj in trajectory classification accuracy. Another important contribution of this work is its uniqueness as the only known US‐based longitudinal investigation of OHRQoL spanning late adolescence to early adulthood.

Several limitations must be acknowledged. First, attrition over the 23+ years of data collection reduced the available sample size at later time points, potentially limiting the statistical power and robustness of our findings. Second, the limited racial and socioeconomic diversity restricts the generalizability of our results to broader populations. Third, the absence of concurrent data on dental caries status is a significant limitation, as it precludes evaluation of the relationship between clinical oral health and perceived quality of life. Future longitudinal studies should aim to incorporate objective clinical measures, including dental caries and other oral health indicators, alongside OHRQoL assessments to enable a more comprehensive understanding of how clinical and subjective oral health evolve together. Moreover, given the strong influence of social determinants on oral health, future research should prioritize inclusion of racially and socioeconomically diverse populations to improve external validity.

We also observed variation in trajectory patterns between CPQ_11‐14_ and GOHR measures, which was anticipated given the conceptual differences between the instruments. The CPQ_11‐14_, designed for youth populations, may be more sensitive to developmental and symptom‐specific changes, while the GOHR offers a broader assessment of overall oral health perceptions. These differences underscore the importance of selecting developmentally appropriate instruments and suggest that a multi‐measure approach may provide a more holistic assessment of OHRQoL across the life course.

Our results carry several important implications. First, the general decline in OHRQoL over time suggests that young adults may face increasing oral health challenges, even if they experienced good OHRQoL in adolescence. This finding highlights the need for targeted interventions to support oral health during this life stage. Second, the association between higher SES and favorable OHRQoL trajectories further reinforces the influence of social determinants on health. These results align with those of Yau et al. [8] and Paula et al. [9], who found that lower‐SES adolescents generally experienced poorer OHRQoL. Collectively, this evidence suggests that reducing socioeconomic disparities, through improved access to dental care and oral health education, may enhance OHRQoL outcomes. However, integrating clinical data into future studies will be essential to fully elucidate biological pathways underlying these associations.

Nevertheless, the limited diversity of our sample calls for cautious interpretation. Most participants were white and from high‐SES backgrounds, which may limit generalizability to more disadvantaged or ethnically diverse populations. Replication in more socioeconomically and racially heterogeneous cohorts is needed to better understand how social determinants influence OHRQoL trajectories.

Several avenues for future research warrant attention. First, longitudinal studies with larger and more diverse samples are needed to validate our findings and enhance external validity. Second, integrating supervised machine learning models may facilitate prediction of trajectory group membership using clinical, behavioral, and psychosocial predictors. This approach could help identify modifiable risk factors and guide targeted interventions. Lastly, extending longitudinal assessments across from early childhood to late adulthood could provide a more complete picture of OHRQoL evolution and identify critical windows for intervention.

## 6 Conclusion

In conclusion, this study contributes to our understanding of how Oral Health‐Related Quality of Life (OHRQoL) evolves from late adolescence into early adulthood. Using longitudinal data from a well‐characterized cohort, we identified distinct subgroups with stable or divergent OHRQoL patterns across three different instruments. While most participants followed trajectories indicative of relatively favorable perceptions, a meaningful proportion experienced persistently lower OHRQoL. The divergence across measures suggests that different instruments may capture unique dimensions of oral health perception, warranting further investigation.

The use of clustering techniques enabled data‐driven exploration of these patterns, though findings should be interpreted cautiously and not taken as definitive classifications. Importantly, socioeconomic status consistently predicted more favorable trajectories, reinforcing the role of social determinants in shaping long‐term oral health experiences. Future research should integrate clinical, behavioral, and psychosocial data to build more comprehensive models of OHRQoL development and inform equitable, targeted preventive strategies during the transition to adulthood.

## Conflicts of Interest

The authors declare no conflicts of interest.

## Supporting information


**Data S1:** Supporting Information.

## Data Availability

The data are not publicly available, but the data that support the findings of this study are available on request from the corresponding author. We are currently working to share all original Iowa Fluoride Study/Iowa Bone Development Study data in 2023 through the dbGaP repository under U01‐DE028522. The code snippet used in performing the machine learning modeling can be found at https://github.com/Drbuxie/OHRQoL‐Trajectory‐1.
