# Supplementary material for: Predicting Group‐Based Trajectories of Oral Health‐Related Quality of Life From Late Adolescence to Early Adulthood Using K‐Means Clustering Algorithm
Source: J Public Health Dent. 2025 Sep 16;85(4):440–8. doi: 10.1111/jphd.70007 (PMC12689277; doi:10.1111/jphd.70007)
Supplement: Supplementary file 1 — Data S1: Supporting Information. [file JPHD-85-440-s001.docx]

APPENDICES

Appendix I

Mean OHRQoL scores at ages 17, 19, 23 and the percentage distributions for the 3-group trajectories

|  | Mean CPQ score (N=374) | | | Percentage distribution | Mean GOHR score (N=374) | | | Percentage distribution | Mean VisQoL score (N=369) | | | Percentage distribution |
| --- | --- | --- | --- | --- | --- | --- | --- | --- | --- | --- | --- | --- |
|  | Age 17 | Age 19 | Age 23 |  | Age 17 | Age 19 | Age 23 |  | Age 17 | Age 19 | Age 23 |  |
| Trajectory group A | 4.38 | 4.74 | 4.93 | 63.6% | 3.35 | 2.75 | 3.02 | 40.6% | 93.11 | 91.60 | 91.22 | 51.5% |
| Trajectory group B | 14.43 | 10.92 | 11.66 | 28.6% | 3.28 | 4.30 | 5.07 | 33.2% | 80.77 | 80.09 | 82.30 | 37.4% |
| Trajectory group C | 29.40 | 27.00 | 25.95 | 7.8% | 5.78 | 5.25 | 5.13 | 26.2% | 75.25 | 63.42 | 56.44 | 11.1% |
| CPQ = Child Perception Questionnaire (possible range = 0 to 148)  GOHR = Global Oral Health Rating (possible range = 2 to 10)  VisQoL = Visual Scoring of Quality of Life (Possible range = 0 to 100) | | | | | | | | | | | | |


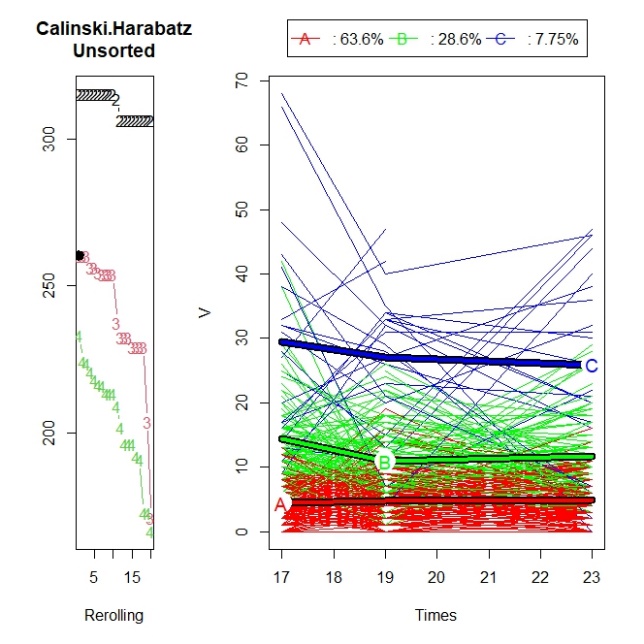
Appendix II

Plot showing the Calinski-Harabatz scores for the different numbers of trajectory groups and individuals’ CPQ trajectories with 3 trajectories grouping of their centroids (for individuals with a maximum of 1 missing time point) (N = 374).

Appendix III


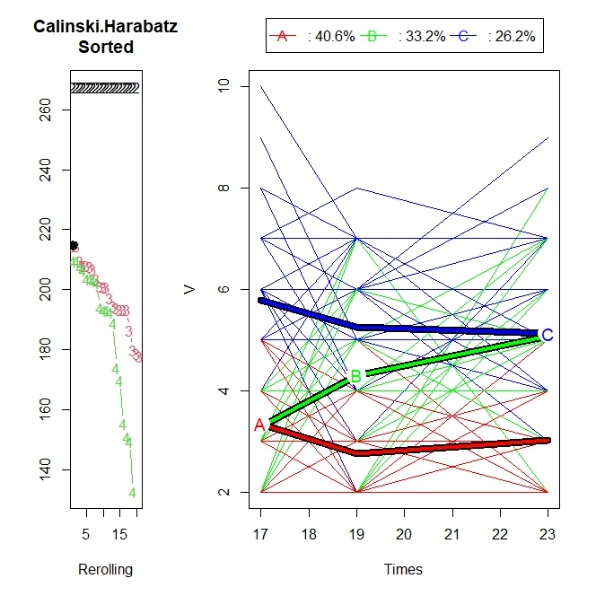


Plot showing the Calinski-Harabatz scores for the different numbers of trajectory groups and individuals’ GOHR trajectories with 3 trajectories grouping of their centroids (for individuals with a maximum of 1 missing time point) (N = 374).

Appendix IV


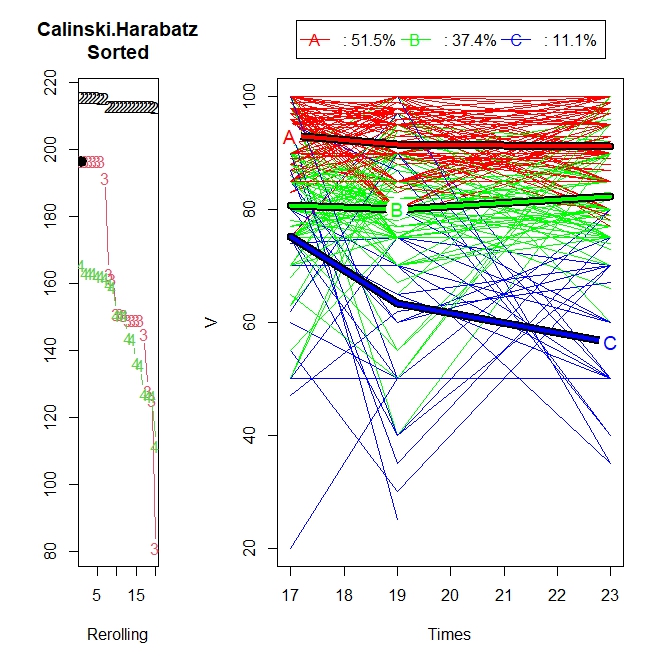


Plot showing the Calinski-Harabatz scores for the different numbers of trajectory groups and individuals’ VisQoL trajectories with 3 trajectories grouping of their centroids (for individuals with a maximum of 1 missing time point) (N = 369).

APPENDIX V

R Code of the trajectory analysis and associative model can be found in my Github repository:

<https://github.com/Drbuxie/OHRQoL-Trajectory-1>
